# Salvia miltiorrhiza (Danshen) promotes Achilles tendon repair: from network pharmacology prediction to in vivo mechanistic validation

**DOI:** 10.3389/fphar.2025.1673962

**Published:** 2025-11-13

**Authors:** Yu Wang, Xianyan Xie, Gaoyuan Yang, Ziyan Li, Shuqi Qin, Junze Luo, Xiaoxu Jia, Yifeng Li, Lin Zhu, Huiguo Wang

**Affiliations:** 1 School of Sport and Health, Guangzhou Sport University, Guangzhou, China; 2 Research Center for Innovative Development of Sports and Healthcare Integration, Guangzhou Sport University, Guangzhou, China; 3 Innovative Research Center for Sports Science in the Guangdong-Hong Kong-Macao Greater Bay Area, Guangzhou Sport University, Guangzhou, China

**Keywords:** Salvia miltiorrhiza, tanshinone IIA, network pharmacology, Achilles tendon repair, regenerative medicine

## Abstract

**Objective:**

To investigate the key bioactive component of Salvia miltiorrhiza (Danshen) for repairing Achilles tendon injury and to elucidate its underlying mechanisms.

**Methods:**

A network pharmacology approach was employed to screen for common targets for Danshen in Achilles tendon repair, identifying Tanshinone IIA (Tan IIA) as the key bioactive component. Its targets were then subjected to Protein-Protein Interaction (PPI) and KEGG pathway analyses. Subsequently, a rat model of Achilles tendon injury was established. After a 4-week intervention with Tan IIA, its reparative effects were comprehensively evaluated using histological, biomechanical, immunohistochemical (IHC), and ELISA methods.

**Results:**

Network pharmacology predicted that Tan IIA acts through core targets such as AKT1 and EGFR to regulate pathways including PI3K-Akt and MAPK. *In vivo* experiments confirmed that, compared to the model group, Tan IIA significantly improved tissue structure (histological score, *P* < 0.001) and enhanced the ultimate tensile load (*P* < 0.01). It also upregulated Collagen Type I (COLⅠ) expression (*P* < 0.05) while downregulating Vascular Endothelial Growth Factor A (VEGFA) expression (*P* < 0.01), and effectively modulated serum inflammation by decreasing IL-6 (*P* < 0.01) while increasing IL-10 and TGF-β (*P* < 0.05).

**Conclusion:**

Tan IIA is the key bioactive component of Salvia miltiorrhiza for repairing Achilles tendon injury. It effectively promotes the structural and functional healing of the tendon by synergistically regulating key processes such as inflammation, matrix remodeling, and angiogenesis.

## 1 Introduction

The Achilles tendon, which connects the gastrocnemius muscle to the calcaneus, is the thickest and strongest tendon in the human body, playing a crucial role in functional movements such as walking, jumping, and running (Kharazi et al., 2021). Due to its limited blood supply and slow metabolism, the Achilles tendon is prone to a series of issues following injury, including impaired structural reconstruction, poor functional recovery, and delayed healing (Al-Dadah and Pal, 2020; Praxitelous et al., 2018). The incidence of Achilles tendon injury is on the rise, particularly among athletes in explosive sports such as basketball, sprinting, and badminton (de Oliveira Júnior et al., 2024). Failure to achieve proper reconstruction after an Achilles tendon injury can not only impair a patient’s normal motor function but also lead to recurrent ruptures and even a complete loss of athletic ability.

Current treatments for Achilles tendon injury primarily involve surgical suture, implantation of artificial scaffolds, and postoperative rehabilitation (Redler, 2022; Seo et al., 2023). Although these strategies can achieve anatomical reconstruction, the repaired tissue often forms a scar-like structure that fails to restore the original biomechanical properties and physiological function. Major challenges in tendon repair persist, including disorganized collagen fiber arrangement in the neotendon, insufficient extracellular matrix (ECM) synthesis, and reduced mechanical strength. Furthermore, the healing process is often complicated by microenvironmental abnormalities such as persistent inflammation, ischemia and hypoxia, fibroblast dysfunction, and insufficient angiogenesis, all of which further compromise the quality of tissue regeneration (Jiang et al., 2023). With recent advances in tissue engineering, targeted drug therapy, and regenerative medicine, researchers are increasingly focusing on strategies to enhance the quality of tendon regeneration and functional recovery. These approaches include modulating cellular behavior, improving the inflammatory microenvironment, and promoting neovascularization (Hou et al., 2025).

In recent years, given the limitations of synthetic drugs in tendon repair, such as potential side effects and single-target specificity, exploring safe and effective therapeutic strategies derived from natural products has emerged as a research hotspot in the field (Adjei-Sowah et al., 2023). Extensive research has demonstrated that various natural active compounds can modulate key processes in tendon repair through multi-target and multi-pathway mechanisms (Li et al., 2025). For instance, curcumin, renowned for its potent anti-inflammatory properties, has been shown to alleviate the early inflammatory response by inhibiting the NF-κB pathway (Córdova et al., 2023), while ginsenoside Rg1 has been found to promote collagen synthesis by upregulating tendon-related growth factors (Wu et al., 2022). These studies collectively highlight the immense potential of natural products in modulating the inflammatory microenvironment, promoting cellular proliferation and differentiation, and optimizing matrix remodeling. Among the numerous candidate natural products, Danshen (Salvia miltiorrhiza), a traditional Chinese herb known for its ability to promote blood circulation and remove blood stasis, has garnered increasing attention for its therapeutic effects on soft tissue injury (Tian et al., 2023), bone fracture healing (He and Shen, 2014), and tendon repair (Li et al., 2025). Modern pharmacological studies have revealed that extracts of Salvia miltiorrhiza possess multiple pharmacological activities, including potent anti-inflammatory, antioxidant, anti-fibrotic, and pro-angiogenic effects. These properties closely align with the complex pathophysiological demands of the tendon injury repair process (Jung et al., 2020). However, in contrast to the growing body of research on isolated active compounds, the material basis and the systemic network of action for a multi-component, multi-target traditional medicine like Salvia miltiorrhiza in the repair of Achilles tendon injury remain to be fully elucidated. Therefore, an in-depth investigation into the mechanisms underlying the therapeutic effects of Salvia miltiorrhiza on Achilles tendon repair is of great significance for the development of novel and effective drugs based on traditional Chinese medicine and for the optimization of treatment strategies for tendon injuries.

This study employed a network pharmacology approach, utilizing multiple databases such as TCMSP, TCMIP, SwissTargetPrediction, GeneCards, and OMIM, to identify the active components, targets, and pathways of Danshen in the context of Achilles tendon injury. We constructed a “drug-component-target-disease” network to screen for the key active components of Danshen responsible for tendon repair. Subsequently, we performed Protein-Protein Interaction (PPI) and Kyoto Encyclopedia of Genes and Genomes (KEGG) pathway analyses on the potential targets of these key components. Finally, by establishing a rat model of Achilles tendon injury and conducting multi-dimensional experimental evaluations, this study aims to comprehensively elucidate the therapeutic potential of Danshen’s key active components from prediction to validation, thereby providing a theoretical and experimental basis for the precise intervention of Danshen in tendon injuries.

## 2 Materials and methods

### 2.1 Screening for key active components of Danshen for Achilles tendon injury treatment

The active components of Danshen and their corresponding targets were retrieved from the Traditional Chinese Medicine Systems Pharmacology Database and Analysis Platform (TCMSP, https://www.tcmsp-e.com), the TCMIP v2.0 (http://www.tcmip.cn), and the Traditional Chinese Medicine Integrated Database (TCMID, https://ngdc.cncb.ac.cn) using “Danshen” as the keyword. For components obtained from TCMSP, a screening was performed based on oral bioavailability (OB) > 30% and drug-likeness (DL) > 0.18. The target protein names were converted to official Gene Symbols using the UniProt database (https://www.uniprot.org/). Finally, the targets from all three databases were merged, and duplicates were removed to generate a final list of active components and targets associated with Danshen.

Targets related to Achilles tendon injury were collected from the GeneCards (https://www.genecards.org) and Online Mendelian Inheritance in Man (OMIM, https://www.omim.org/) databases using “Achilles tendon injury” and “Injury of Achilles tendon” as keywords. The targets obtained from both databases were then merged, and duplicates were removed to create a comprehensive list of disease-related targets.

The target lists for “Danshen” and “Achilles tendon injury” were imported into the Venny 2.1.0 online tool (https://bioinfogp.cnb.csic.es/tools/venny/) to generate a Venn diagram and identify the potential common targets for Danshen in the treatment of Achilles tendon injury.

The relationships among Danshen, its active components, the potential targets, and Achilles tendon injury were visualized by constructing a “drug-component-target-disease” network using Cytoscape software (version 3.10.0). The Centiscape 2.2 plugin was used to calculate topological parameters including Degree, Betweenness, and Closeness. Based on these values, the key active components of Danshen for repairing Achilles tendon injury were identified.

### 2.2 Network pharmacology-based validation of the key active components

The predicted targets of the key active components identified in the previous step were retrieved from the SwissTargetPrediction database (http://swisstargetprediction.ch) using the names of the key components as queries.

A Venn diagram was generated using Venny 2.1.0 to identify the overlapping targets between the predicted targets of the “key active components” and the “Achilles tendon injury”-related targets obtained in section 2.1. These overlapping targets were considered the potential targets for the key active components in treating Achilles tendon injury.

The potential targets were imported into the STRING database (https://cn.string-db.org/) to construct a protein-protein interaction (PPI) network and obtain interaction data. The interaction data was then imported into Cytoscape (version 3.10.0), and the Centiscape 2.2 plugin was used to calculate the Degree, Betweenness, and Closeness values. The top 10 proteins, based on these metrics, were identified as core targets.

The core targets were then subjected to pathway enrichment analysis using the Kyoto Encyclopedia of Genes and Genomes (KEGG) database (https://www.genome.jp/kegg/) to identify the key signaling pathways through which the key active components of Danshen may repair Achilles tendon injury.

### 2.3 Therapeutic effects and mechanisms of Tan IIA on Achilles tendon injury

#### 2.3.1 Experimental animals and grouping

Experimental animals were purchased from the Experimental Animal Center of Southern Medical University (License No. SCXK [Yue] 2021-0041) and housed in a standard animal facility. All animal experiments were approved by the Ethics Committee of Guangzhou Sport University (Approval No. 2023LCLL-87) and were conducted in strict accordance with the guidelines of the Animal Care and Use Committee.

A total of 45 male Sprague-Dawley (SD) rats (8 weeks old, weighing 220 ± 20 g) were randomly divided into three groups (n = 15 per group): a sham-operated group (Sham), an Achilles tendon injury model group (Model), and a Tan IIA treatment group (Tan IIA). The intervention period was 4 weeks.

Model Preparation: The Sham group served as a negative control, in which animals underwent only a skin incision without any injury to the Achilles tendon, to rule out the effects of the surgical procedure itself. In the Model and Tan IIA groups, the rat model of Achilles tendon injury was established according to a previously described method (Tuncer et al., 2021). Briefly, after a 12-h fast, rats were anesthetized with an intraperitoneal injection of a mixture of 5% chloral hydrate and 12.5% urethane (5 mL/kg). Once anesthetized, the right hind limb was shaved, disinfected, and the rat was placed in a supine position on a heated surgical table. To induce the injury, a longitudinal incision of approximately 10 mm was made proximal to the Achilles tendon insertion to expose the tendon. The tendon was then transected transversely approximately 5 mm from its calcaneal insertion to simulate a full-thickness rupture. The severed tendon and skin were subsequently sutured using the modified Kessler suture technique. All animals received post-operative care. After recovering from anesthesia, they were treated with penicillin (20,000 U/day for 3 consecutive days) as an anti-infective measure to reduce inflammation and alleviate secondary pain associated with potential infection. Furthermore, we monitored the animals daily for food intake, activity levels, and wound healing. For any individual exhibiting overt signs of pain (such as a persistent huddled posture or impaired mobility), supplementary oral analgesia with acetaminophen was administered in accordance with the guidelines of our Ethics Committee.

Intervention: For 4 weeks, the SHAM and Model groups were administered normal saline by oral gavage. The Tan IIA group received daily oral gavage of Tan IIA at a dose of 15 mg/kg/day. The gavage volume for all groups was 3 mL/kg. Throughout the experiment, the animals were maintained on a 12-h light/dark cycle. The dark cycle was from 20:00 to 08:00, during which food was withdrawn. The light cycle was from 08:00 to 20:00, during which food was provided. Water was available *ad libitum* at all times.

#### 2.3.2 Biomechanical testing

After 4 weeks of intervention, seven rats were randomly selected from each group for biomechanical testing of the Achilles tendon. Animals were deeply anesthetized using the same method as for the surgery (intraperitoneal injection of a mixture of 5% chloral hydrate and 12.5% urethane) and subsequently euthanized by an overdose of this anesthetic. Death was confirmed by the complete cessation of heartbeat and respiration, as well as the absence of the pedal reflex. The right hind limb was dissected, and surrounding muscle tissues were carefully removed, preserving the entire Achilles tendon-calcaneus complex. The specimens were wrapped in sterile gauze soaked in normal saline and prepared for ultimate tensile load testing. For the test, the tendon-calcaneus complex was mounted on the clamps of a universal testing machine (Instron 5,967/Z 100, Instron, United States). A tensile load was applied along the longitudinal axis of the tissue at a constant speed of 0.1 mm/s. The maximum tensile force at the point of rupture was recorded as the ultimate tensile load.

#### 2.3.3 Histological staining

The remaining rats in each group were used for histological analysis. After anesthesia and euthanasia, the tissue segment from the mid-calf to the mid-foot, including the relevant structures, was harvested. The samples were fixed in neutral buffered formalin for 24 h, followed by standard procedures for dehydration, clearing, paraffin embedding, and sectioning into 4 μm-thick slices.

Histopathological Staining: Tissue sections were stained with Hematoxylin and Eosin (H&E) to observe cell morphology and tissue arrangement, and with Masson’s trichrome staining to evaluate collagen fiber deposition. To systematically evaluate tendon repair, a semi-quantitative analysis was performed using the Bonar histological grading scale (Aydın et al., 2015). This scale assesses five parameters: tenocyte morphology, tenocyte proliferation, collagen bundle alignment, vascularity, and ground substance, with each parameter scored from 0 to 3. A higher total score indicates poorer tissue repair quality.

Immunohistochemical (IHC) Staining: Standard immunohistochemical methods were used to detect the expression levels of Collagen Type I (Col I; Anti-Collagen I antibody, Abcam, United Kingdom) and Vascular Endothelial Growth Factor A (VEGF-A; VEGFA Polyclonal antibody, Proteintech Group, United States). Tissue sections were deparaffinized and rehydrated. Antigen retrieval was performed by heating the slides in an EDTA buffer (pH 9.0) under high pressure. After cooling to room temperature, endogenous peroxidase activity was blocked with 3% H_2_O_2_. The sections were then incubated with 10% normal serum at 37 °C for 30 min. Primary antibodies (COL1, VEGF-A) were diluted according to the manufacturer’s instructions, applied to the sections, and incubated overnight at 4 °C. The following day, after washing three times with TBST, a diluted HRP-conjugated secondary antibody was added and incubated at 37 °C for 45 min. The color was developed with a DAB substrate kit, monitored under a microscope, and the reaction was terminated with tap water. Nuclei were counterstained with hematoxylin, differentiated with acid alcohol, and blued. Finally, the sections were dehydrated, dried, and mounted. All sections were observed under a microscope, and images were captured for analysis. The average optical density was quantified using ImageJ software.

#### 2.3.4 Serum biochemical analysis

Blood samples from all experimental animals were collected via the jugular vein to obtain serum. After anesthesia, the clavicular region was shaved, and the animal was secured in a supine position with its head turned to expose the jugular vein. Blood was drawn using a 1 mL syringe. The collected blood was allowed to stand at 4 °C for 2 h and then centrifuged at 3,000 rpm for 10 min to separate the serum. The serum levels of IL-6, IL-10, and TGF-β were measured using Enzyme-Linked Immunosorbent Assay (ELISA) kits (Key-Bio, Nanjing, China).

### 2.4 Statistical analysis

All data were analyzed using SPSS software (version 26.0). The normality of data distribution was assessed using the Shapiro-Wilk test, and the homogeneity of variances was evaluated with Levene’s test. For data that met the assumptions of normality and homogeneity of variance, one-way analysis of variance (ANOVA) followed by Tukey’s post-hoc test was used. Otherwise, the Kruskal-Wallis non-parametric test was employed. A P-value <0.05 was considered statistically significant. Image analysis was performed using Fiji-ImageJ software (version 1.54), and graphs were generated with GraphPad Prism software (version 10.0).

## 3 Results

### 3.1 Identification of core targets of Danshen in Achilles tendon injury

**FIGURE 1:**
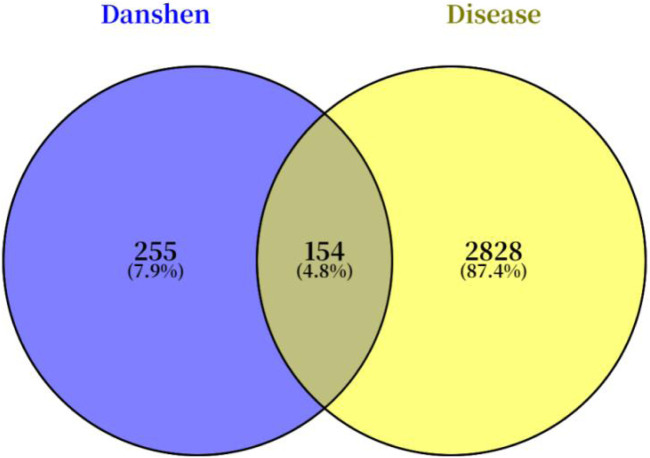
Potential therapeutic targets of Salvia miltiorrhiza for Achilles tendon injury.

A total of 56 bioactive components and 409 potential targets of Salvia miltiorrhiza were identified by searching the TCMSP, TCMIP, and TCMID databases. Concurrently, a total of 2,986 targets associated with Achilles tendon injury were retrieved from the GeneCards and OMIM databases. By intersecting the targets of Danshen with those of Achilles tendon injury, 154 potential therapeutic targets were identified, as illustrated in the Venn diagram (Figure 1).

### 3.2 Identification of key active compounds

**FIGURE 2:**
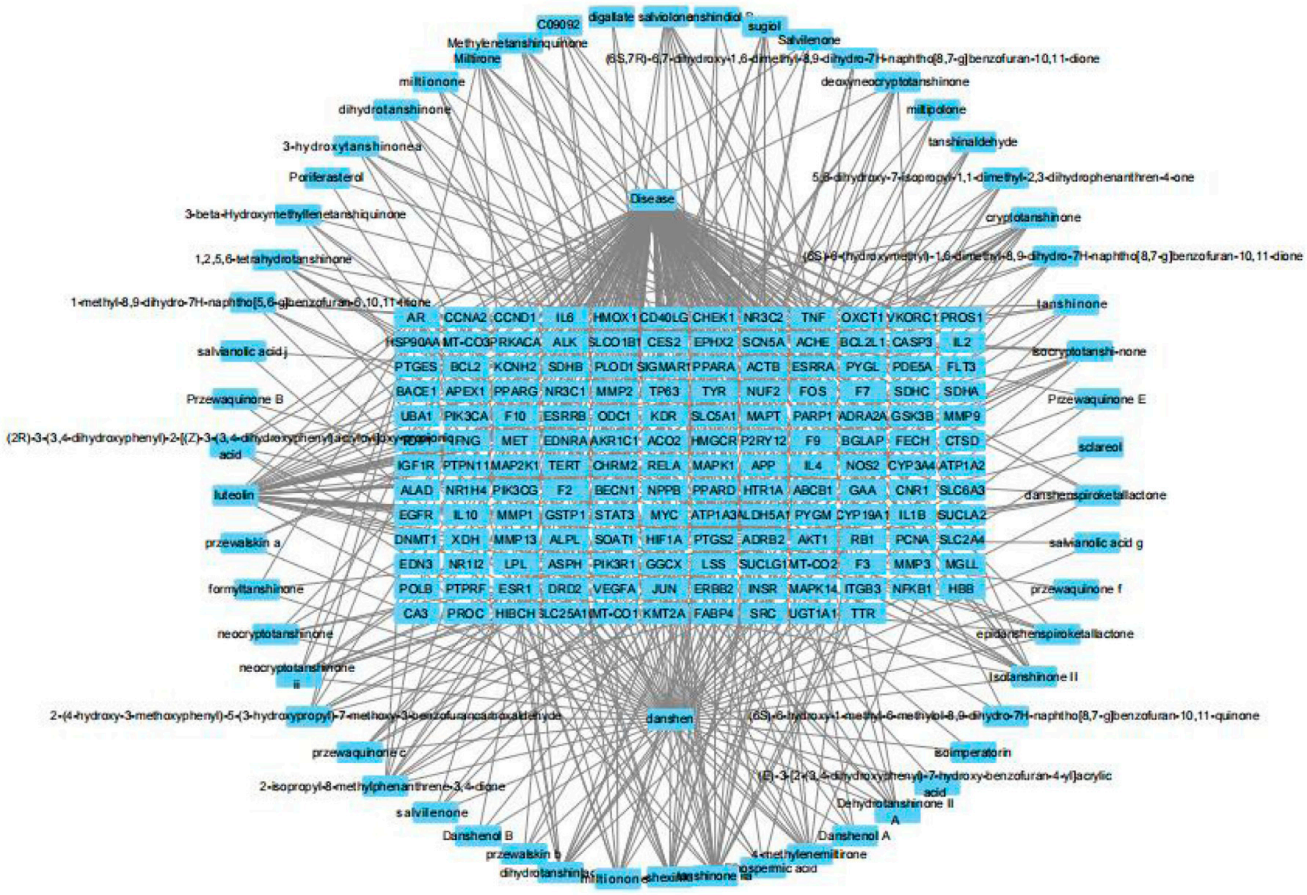
The “drug-component-target-disease” network of Salvia miltiorrhiza and Achilles tendon injury.

The active components of Danshen and the 154 potential targets identified in section 3.1 were imported into Cytoscape 3.10.0 to construct a “drug-component-target-disease” network. As shown in Figure 2, Danshen acts on Achilles tendon injury through multiple components and multiple targets.

The Centiscape 2.2 plugin was used to calculate the Degree, Betweenness, and Closeness values for the active components in the network. As shown in Table 1, the top five active components of Danshen acting on Achilles tendon injury were Luteolin, Tan IIA, Cryptotanshinone, Dan-shexinkum D, and Isotanshinone II.

**TABLE 1:** Top five key bioactive components of Salvia miltiorrhiza for repairing Achilles tendon injury.

Name	Betweenness unDir	Closeness unDir	Degree unDir
luteolin	1937.345575	0.001980198	36
Tan IIA	767.9432038	0.001855288	19
cryptotanshinone	266.2304097	0.001801802	11
dan-shexinkum d	187.6272853	0.001814882	13
Isotanshinone II	129.4906509	0.001808318	12

Although luteolin ranked highest in the network topological analysis, it is a common flavonoid found in many plants and is not a unique bioactive constituent of Danshen. In contrast, Tan IIA is a signature and specific active component of the Salvia genus. Its high content and lipophilic nature form the material basis for its pharmacological effects. Therefore, Tan IIA was selected for subsequent experimental investigation into its role in Achilles tendon repair.

### 3.3 Potential targets of Tan IIA for Achilles tendon repair

**FIGURE 3:**
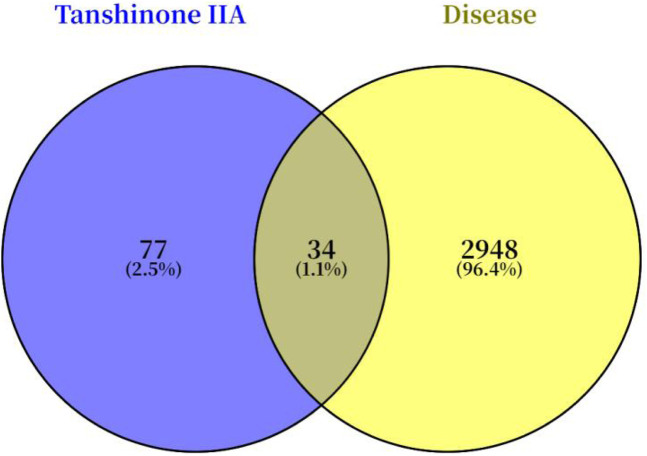
Overlapping targets of Tan IIA in Achilles tendon injury.

A search for “Tanshinone IIA” in the SwissTargetPrediction database yielded 110 predicted targets. A Venn diagram was constructed to compare these predicted targets with the Achilles tendon injury-related targets identified in section 3.1 (Figure 3), which revealed 34 potential common targets for Tan IIA in repairing Achilles tendon injury.

### 3.4 PPI network analysis and core target identification for Tan IIA

**FIGURE 4:**
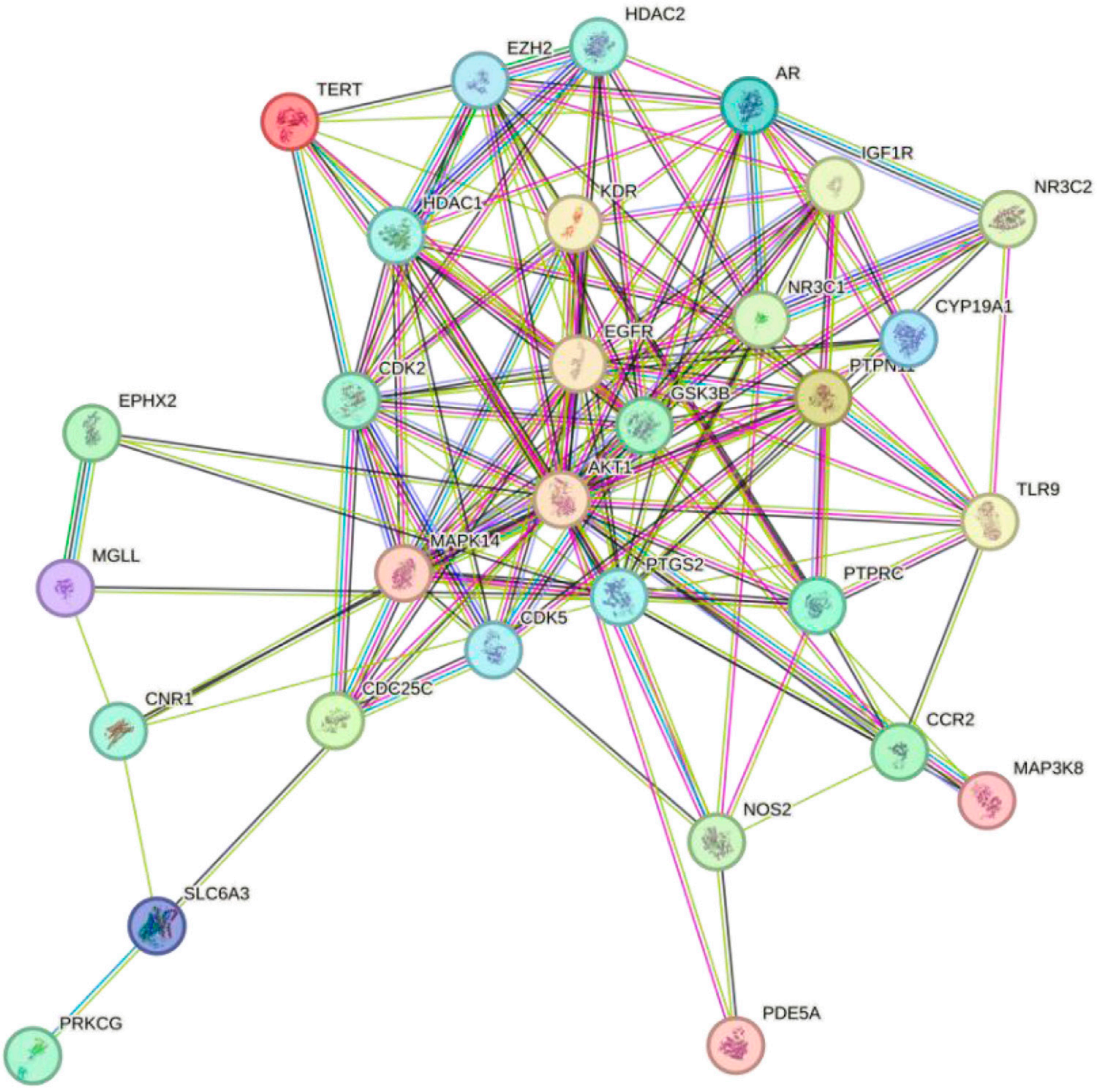
PPI network of Tan IIA’s potential targets for Achilles tendon repair.

To investigate the interactions among the potential targets of Tan IIA in Achilles tendon repair, the 34 potential targets from section 3.3 were imported into the STRING database to construct a PPI network (Figure 4). The network consisted of 34 nodes and 140 edges, indicating close biological connections among these proteins.

To identify the core regulatory targets of Tan IIA in Achilles tendon repair, the PPI network data was imported into Cytoscape, and the topological parameters of each node were calculated using the Centiscape 2.2 plugin. Based on the Degree, Betweenness, and Closeness values, the top ten core targets were identified as AKT1, EGFR, PTGS2, MAPK14, CDK2, GSK3B, AR, EZH2, HDAC1, and NR3C1 (Table 2).

**TABLE 2:** Top ten core therapeutic targets of Tan IIA for Achilles tendon repair.

Name	Degree unDir	Betweenness unDir	Closeness unDir
AKT1	52	270.700	0.031
EGFR	42	68.165	0.026
PTGS2	40	109.517	0.026
MAPK14	28	23.918	0.022
CDK2	28	20.087	0.022
GSK3B	28	15.546	0.022
AR	28	17.018	0.022
EZH2	24	7.192	0.021
HDAC1	24	10.621	0.021
NR3C1	22	10.046	0.021

### 3.5 Pathway analysis of Tan IIA in Achilles tendon injury

To elucidate the molecular mechanisms of Tan IIA in Achilles tendon repair, a KEGG pathway enrichment analysis was performed on the 34 potential targets identified in section 3.3. A total of 92 significant pathways were enriched (P < 0.05). Based on the key pathophysiological processes of tendon repair, we selected and categorized 17 of these pathways related to cell proliferation, inflammation, angiogenesis, and extracellular matrix (ECM) remodeling.

**FIGURE 5:**
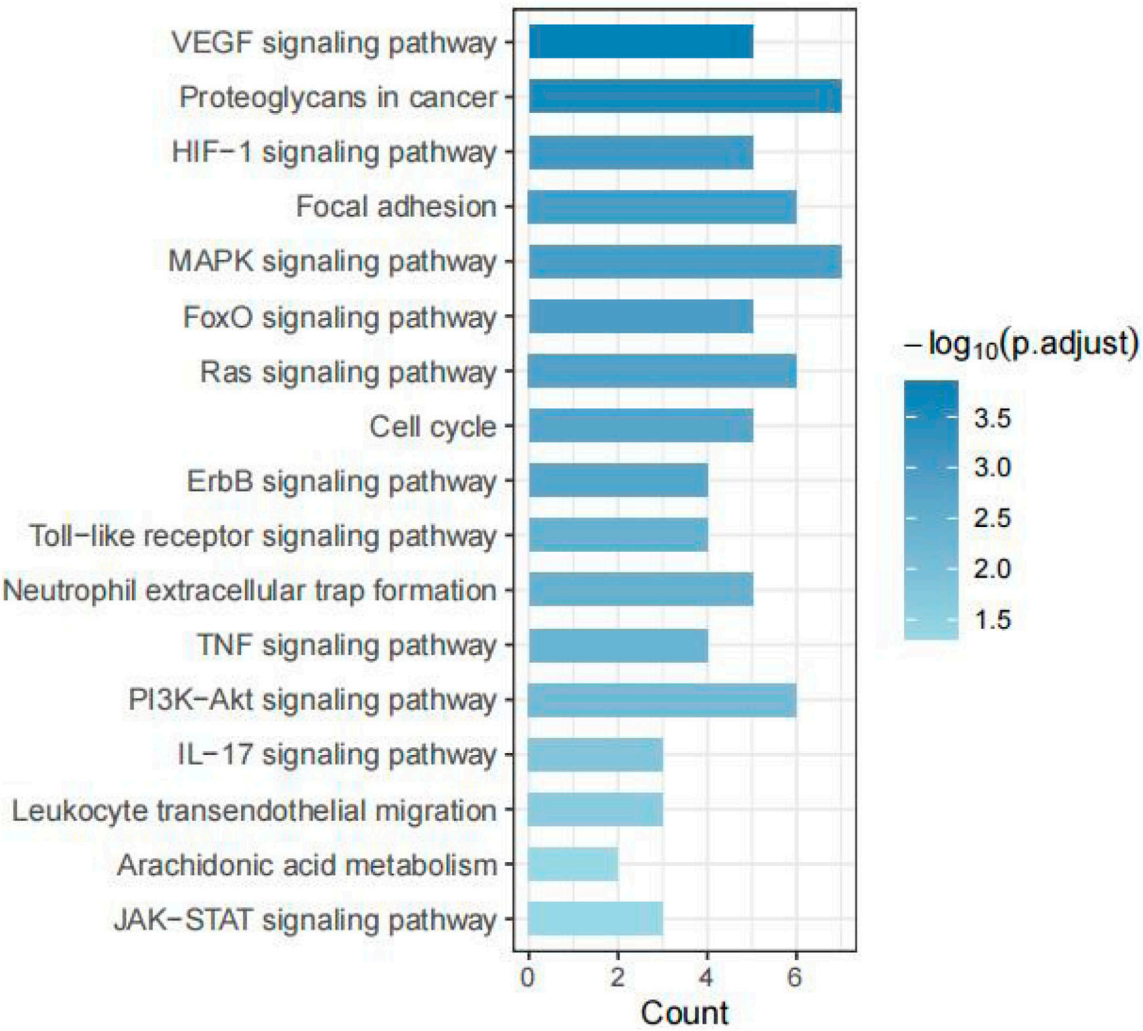
KEGG pathway enrichment analysis of Tan IIA’s targets in Achilles tendon repair.

As shown in Figure 5, 17 pathways were highly relevant to Achilles tendon repair. These included pathways related to cell proliferation, survival, and differentiation (PI3K-Akt signaling pathway, MAPK signaling pathway, Ras signaling pathway, ErbB signaling pathway, Cell cycle, FoxO signaling pathway); inflammation and immune response (TNF signaling pathway, Toll-like receptor signaling pathway, JAK-STAT signaling pathway, IL-17 signaling pathway, Arachidonic acid metabolism, Leukocyte transendothelial migration, Neutrophil extracellular trap formation); angiogenesis (VEGF signaling pathway, HIF-1 signaling pathway); and cell adhesion and matrix remodeling (Focal adhesion, Proteoglycans in cancer).

### 3.6 Tan IIA promoted structural reconstruction after Achilles tendon injury

**FIGURE 6:**
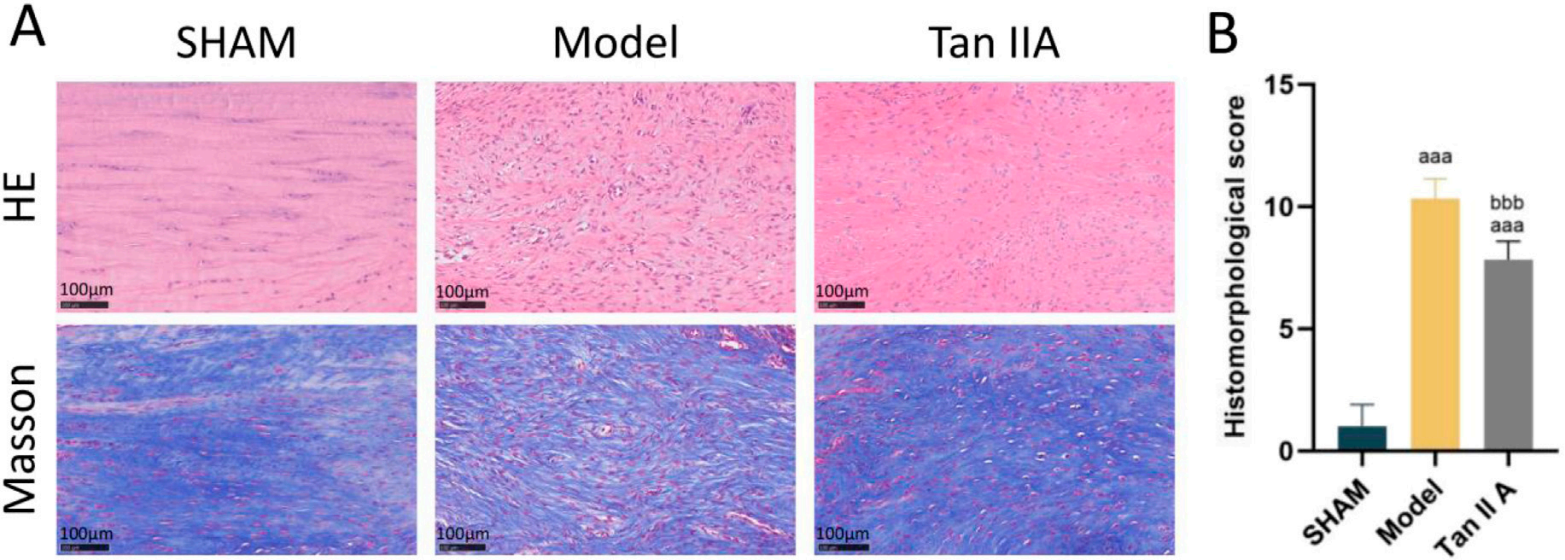
**(A)** Representative histological images of rat Achilles tendon tissue at 4 weeks post-surgery stained with H&E (upper row) and Masson’s trichrome (lower row). **(B)** Quantitative histomorphological scores based on Bonar’s scale. Note: a, aa, aaa indicate *P* < 0.05, *P* < 0.01, *P* < 0.001, respectively, compared with the SHAM group; b, bb, bbb indicate *P* < 0.05, *P* < 0.01, *P* < 0.001, respectively, compared with the Model group.

The results of HE and Masson’s trichrome staining are shown in Figure 6A. In the Sham group, the Achilles tendon tissue exhibited an intact structure. HE staining revealed well-organized, spindle-shaped tenocytes with slender, regularly distributed nuclei, and the tissue structure was dense. Masson’s staining showed regularly arranged, parallel, wavy collagen fibers with uniform blue staining and a compact structure, consistent with normal tendon morphology. In the Model group, there was significant tissue disruption. H&E staining showed disordered tenocyte arrangement, rounded nuclei, and marked inflammatory cell infiltration. Masson’s staining revealed separated and disorganized collagen bundles with reduced staining intensity, indicating a loose tissue structure and poor healing quality. The histological appearance of the Tan IIA group showed marked improvement compared to the Model group. In H&E stains, tenocytes appeared more elliptical or spindle-shaped with a more consistent alignment, and the number of inflammatory cells was reduced. Masson’s staining showed more orderly and densely packed collagen bundles with enhanced blue staining, suggesting improved collagen deposition and remodeling.

The semi-quantitative histological scoring results (Figure 6B) showed that the total histological scores of both the Model and Tan IIA groups were extremely significantly higher than that of the Sham group (P < 0.001). Compared to the Model group, the total histological score of the Tan IIA group was extremely significantly lower (P < 0.001). These results indicate that Tan IIA has a positive effect on promoting structural reconstruction and tissue repair of the Achilles tendon.

### 3.7 Tan IIA enhanced the biomechanical recovery after Achilles tendon injury

**FIGURE 7:**
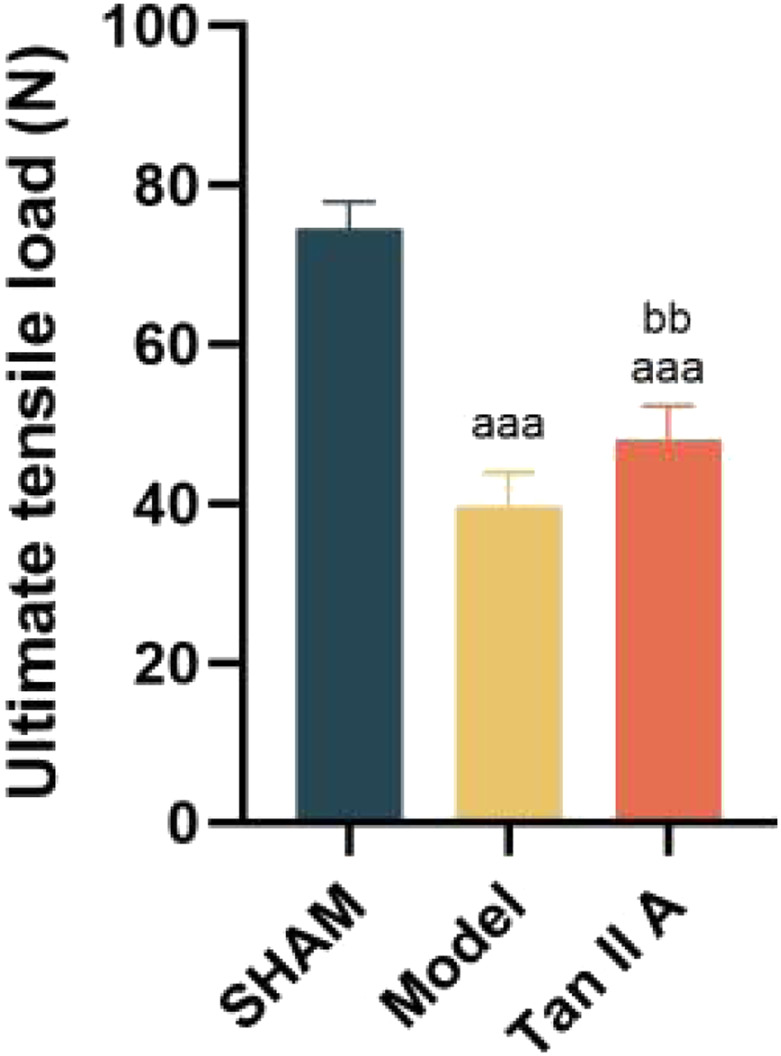
Comparison of ultimate tensile load of rat Achilles tendon tissue at 4 weeks post-surgery. Note: a, aa, aaa indicate *P* < 0.05, *P* < 0.01, *P* < 0.001 respectively, compared with the SHAM group. b, bb, bbb indicate *P* < 0.05, *P* < 0.01, *P* < 0.001 respectively, compared with the Model group.

The results of the ultimate tensile load test are shown in Figure 7. Compared to the Sham group, the ultimate tensile load of both the Model and Tan IIA groups was extremely significantly lower (P < 0.001). However, the Tan IIA group exhibited a very significantly higher ultimate tensile load compared to the Model group (P < 0.01). These findings demonstrate that Tan IIA intervention can effectively improve the ultimate tensile load of the injured tendon, thereby promoting tissue repair.

### 3.8 Tan IIA promoted protein synthesis after Achilles tendon injury

**FIGURE 8:**
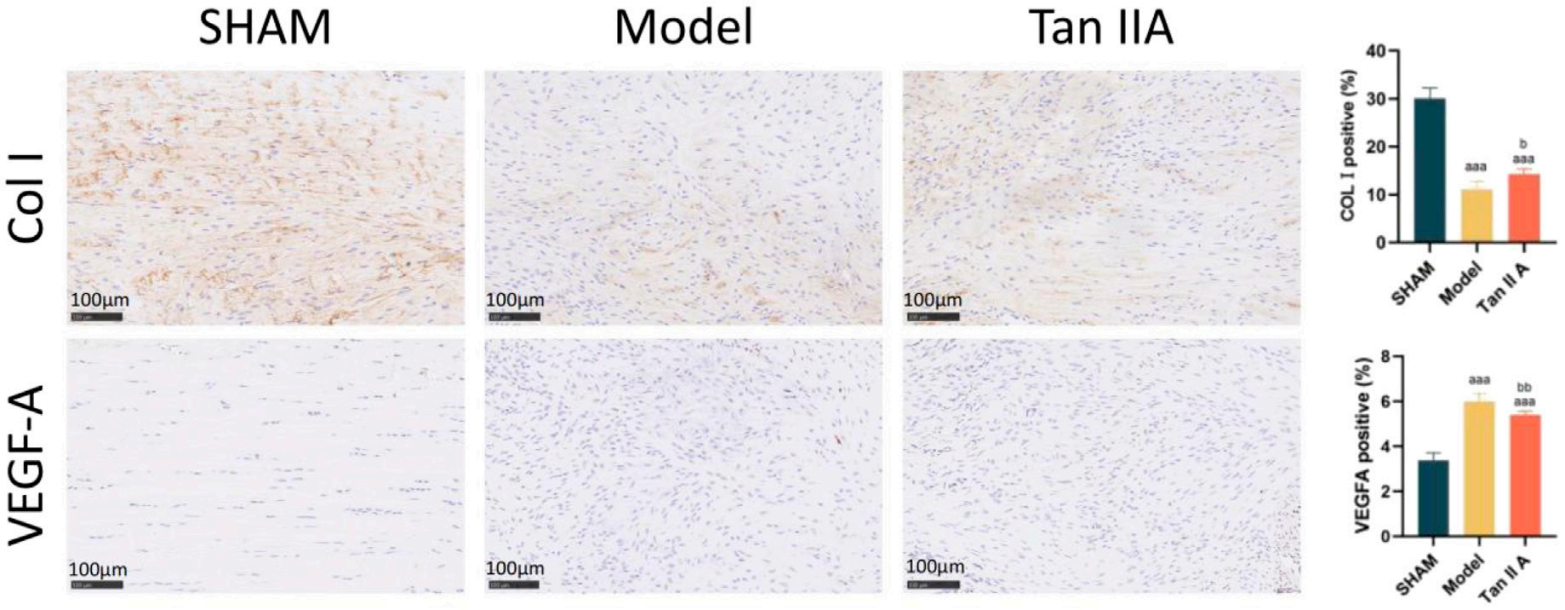
Immunohistochemical staining and semi-quantitative analysis of rat Achilles tendon tissue at 4 weeks post-surgery. Note: a, aa, aaa indicate *P* < 0.05, *P* < 0.01, *P* < 0.001 respectively, compared with the SHAM group. b, bb, bbb indicate *P* < 0.05, *P* < 0.01, *P* < 0.001 respectively, compared with the Model group.

The results of immunohistochemical staining of the Achilles tendon tissue are shown in Figure 8.

Regarding COL I expression: The percentage of COL I-positive area was extremely significantly lower in both the Model and Tan IIA groups compared to the Sham group (P < 0.001). Compared to the Model group, the Tan IIA group showed a statistically significant increase in COL I-positive expression (P < 0.05).

Regarding VEGFA expression: The percentage of VEGFA-positive area was extremely significantly higher in both the Model and Tan IIA groups compared to the Sham group (P < 0.001). However, compared to the Model group, the Tan IIA group exhibited a very significantly lower level of VEGFA-positive expression (P < 0.01).

In summary, these findings suggest that Tan IIA treatment can, to some extent, enhance COL I synthesis, promoting tissue matrix reconstruction, while also modulating VEGFA expression.

### 3.9 Tan IIA regulated the inflammatory microenvironment after Achilles tendon injury

**FIGURE 9:**
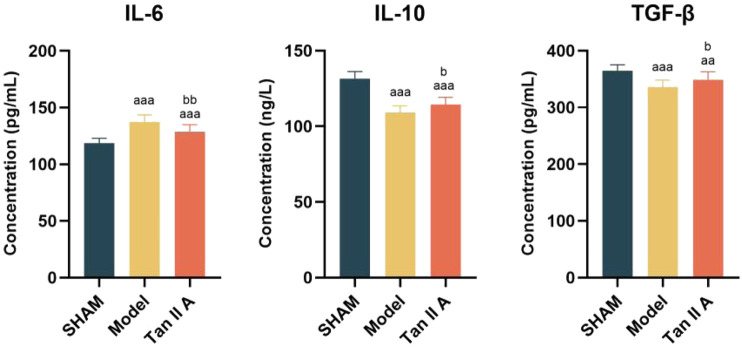
Comparison of serum levels of IL-6, IL-10, and TGF-β in rats at 4 weeks post-surgery. Note: a, aa, aaa indicate *P* < 0.05, *P* < 0.01, *P* < 0.001 respectively, compared with the SHAM group. b, bb, bbb indicate *P* < 0.05, *P* < 0.01, *P* < 0.001 respectively, compared with the Model group.

The changes in serum levels of IL-6, IL-10, and TGF-β in the different experimental groups are shown in Figure 9.

Regarding IL-6: Compared to the Sham group, IL-6 levels were extremely significantly elevated in both the Model and Tan IIA groups (P < 0.001). Compared to the Model group, the IL-6 level in the Tan IIA group was very significantly decreased (P < 0.01). This indicates that Tan IIA intervention can effectively suppress the abnormal increase in the pro-inflammatory cytokine IL-6.

Regarding IL-10: Compared to the Sham group, IL-10 levels were extremely significantly lower in both the Model and Tan IIA groups (P < 0.001). However, the Tan IIA group showed a statistically significant increase in IL-10 levels compared to the Model group (P < 0.05). This result suggests that Tan IIA intervention enhances the expression of the anti-inflammatory cytokine IL-10, contributing to the resolution of inflammation.

Regarding TGF-β: Compared to the Sham group, the TGF-β expression level was extremely significantly lower in the Model group (P < 0.001) and very significantly lower in the Tan IIA group (P < 0.01). Compared to the Model group, the TGF-β level was statistically significantly higher in the Tan IIA group (P < 0.05). This suggests that Tan IIA intervention can promote the upregulation of the pro-repair factor TGF-β.

In summary, Tan IIA intervention modulates the systemic inflammatory microenvironment by downregulating IL-6 and upregulating IL-10 and TGF-β, thereby creating a more favorable biological condition for Achilles tendon repair.

## 4 Discussion

The repair of Achilles tendon injury is a dynamic and complex biological process involving the synergistic action of multiple mechanisms, including cell proliferation, migration, extracellular matrix (ECM) remodeling, angiogenesis, and inflammatory response regulation (Schulze-Tanzil et al., 2022). Although conventional clinical surgery and rehabilitation methods can restore the structure and function of tendon tissue to a certain extent, the quality of postoperative repair is often compromised by factors such as persistent local inflammation, disordered collagen fiber alignment, and an unfavorable microenvironment. These issues lead to a high risk of re-injury and poor functional recovery. Therefore, developing pharmacological interventions with well-defined targets capable of modulating the tissue repair microenvironment is a key direction for enhancing the efficacy of Achilles tendon repair.

In this study, by constructing a ‘drug-component-target-disease’ network and integrating the pharmacological properties, content within Salvia miltiorrhiza, and existing literature support, we ultimately identified Tan IIA as the key active component responsible for the tendon repair-promoting effects of Danshen. Although luteolin ranked highest in the network topological analysis, it is widely present in many plants and is not a specific component of S. miltiorrhiza. In contrast, Tan IIA is a characteristic component of Danshen, and previous studies have confirmed its anti-inflammatory and tissue-reparative activities, making it a more suitable subject for investigating the mechanisms by which Danshen promotes Achilles tendon repair.

This study systematically revealed that Tan IIA may regulate Achilles tendon injury repair through a complex network involving multiple targets and pathways. The ten core targets identified through network topological analysis, including AKT1, EGFR, and PTGS2, are all key factors in the field of tissue injury and repair. AKT1 is a central node in the PI3K-Akt signaling pathway, which has been confirmed as a crucial hub for regulating tenocyte proliferation, differentiation, and anti-apoptosis (Goto et al., 2025). The targeted activation of AKT1 by Tan IIA may directly promote the proliferation of tendon stem/progenitor cells (TSPCs), providing an ample cell source for tissue reconstruction. Activation of EGFR can initiate multiple downstream signaling cascades, including the Ras-MAPK pathway, to promote cell migration and proliferation, which is essential for filling the Achilles tendon defect (Chen et al., 2024). PTGS2 is a key rate-limiting enzyme in the arachidonic acid metabolic pathway and a primary mediator of the inflammatory response. The regulatory effect of Tan IIA on PTGS2 suggests that it may create a favorable microenvironment for repair in the early stages of injury by inhibiting excessive inflammation, which is consistent with the findings of Zhang regarding Tan IIA’s inhibition of inflammation via the MAPK pathway (Zhang et al., 2023). KEGG enrichment analysis further integrated these discrete targets into a functional signaling pathway network, identifying 17 highly relevant pathways based on the key pathological processes of tendon injury repair. These pathways can be categorized into four key processes in Achilles tendon repair: inflammation regulation, cell proliferation, angiogenesis, and tissue remodeling, indicating that Tan IIA does not act on a single step. In the early phase of injury, Tan IIA may inhibit excessive inflammatory responses by regulating pathways such as TNF, Toll-like receptor, and JAK-STAT, thereby clearing obstacles and creating a favorable microenvironment for the subsequent repair phases. As repair progresses, Tan IIA synergistically drives tenocyte proliferation and neovascularization by activating key pathways like PI3K-Akt, MAPK, VEGF, and HIF-1, supplying essential oxygen and nutrients to the repair zone. Finally, by influencing the Focal adhesion pathway, Tan IIA may promote the orderly alignment of newly formed collagen fibers and enhance their connection with cells—a critical step in transforming fragile granulation tissue into a tough, load-bearing neotendon.

This study further validated the tissue repair effects of Tan IIA in Achilles tendon injury by establishing a rat model. To ensure the scientific validity and efficacy of the *in vivo* experiments, we adopted a dose of 15 mg/kg/day. This dosage was selected in reference to previously reported effective oral gavage doses of Tan IIA in rodent models (Xu et al., 2025), while also taking into account its pharmacokinetic properties and considerations for interspecies body surface area scaling (Reagan-Shaw et al., 2008). This approach ensures that the compound can exert sufficient pharmacological effects at the animal level without causing adverse toxic effects. Furthermore, the calculated Human Equivalent Dose is consistent with conventional clinical dosage ranges, thereby strengthening the scientific rationale and clinical translational potential of our findings. At the histological level, Tan IIA demonstrated a significant promoting effect on tissue repair. Hematoxylin and eosin (HE) staining results showed that Tan IIA intervention significantly ameliorated pathological features in the Achilles tendon injury model group, such as disordered tenocyte arrangement and rounded nuclei. The cells recovered their typical spindle shape and gradually aligned in a uniform direction, suggesting a positive role for Tan IIA in regulating cell behavior and promoting structural reconstruction. Masson’s trichrome staining further confirmed the effects on matrix remodeling. In the Tan IIA-treated group, collagen fibers were denser and more regularly arranged, with enhanced staining intensity, indicating that the orderly deposition and structural remodeling of collagen were effectively promoted. This finding is highly consistent with that of Zhou et al. in a rat Achilles tendon repair model, where they demonstrated that a combination therapy involving Tan IIA improved the orderly alignment of type I collagen fibers by regulating the TGF-β/Smad3 signaling pathway (Zhou et al., 2020). Furthermore, the Bonar scores revealed that the Tan IIA group was superior to the model group across multiple dimensions, including tenocyte morphology, collagen bundle alignment, and vascularity, providing further semi-quantitative evidence of its overall promoting effect on tendon tissue repair.

As a biological tissue responsible for transmitting mechanical forces, the ultimate tensile load of the Achilles tendon directly reflects its structural integrity and the level of functional healing (Schulze-Tanzil et al., 2022). Our results show that Tan IIA intervention significantly increased the ultimate tensile load of the injured Achilles tendon, providing direct evidence of its ability to promote functional recovery. This improvement in mechanical properties is not an isolated phenomenon but is closely related to the orderly rearrangement and densification of collagen fibers observed histologically. Notably, some studies report that certain natural compounds, while increasing collagen deposition, fail to significantly improve the tissue’s tensile strength (Gemalmaz et al., 2018). This is typically attributed to the haphazard arrangement of nascent collagen fibers, which prevents the formation of an effective load-bearing network. In contrast, this study confirms that Tan IIA synergistically promotes both the “quantity” (increased deposition) and “quality” (optimized alignment) of collagen. By facilitating orderly structural reconstruction, it achieves a synchronous improvement in tissue morphology and mechanical function, which is of crucial clinical significance for preventing re-rupture after repair.

Type I collagen (COL I) is the main component of tendons (approximately 80%–90%), and its expression level directly determines the mechanical strength of the tissue (Benage et al., 2022). The immunohistochemistry results in our study clearly showed that, compared to the model group, COL I expression was significantly upregulated in the Tan IIA-treated group. This finding not only provides a molecular-level explanation for the aforementioned histological and mechanical improvements but also aligns perfectly with the predicted activation of signaling pathways such as PI3K-Akt and TGF-β from our network pharmacology analysis. Previous research has established that the PI3K-Akt pathway is a key hub for regulating tenocyte proliferation and matrix synthesis, while the TGF-β signal is one of the most classic and potent inducers of COL I gene transcription. Therefore, it is highly probable that Tan IIA drives tenocytes to synthesize more high-quality COL I by targeting and activating these upstream pathways, thereby providing a sufficient ECM for a robust neotendon.

This study also observed a finding that may seem paradoxical but is consistent with the physiological rhythm of tendon repair: at the mid-to-late stage of repair (4 weeks), the expression of VEGF-A in the Tan IIA group was significantly lower than in the model group (P < 0.01). This result should be understood in the context of the biphasic role of VEGF-A in tendon healing. In the early stages (inflammatory and proliferative phases, approx. 1–3 weeks), VEGF-A-mediated angiogenesis is indispensable for supplying nutrients and oxygen and for recruiting inflammatory and stem cells, making it a critical step for initiating repair (Buettmann et al., 2019). However, upon entering the remodeling phase (approx. after 4 weeks), persistently high levels of VEGF-A can lead to sustained neovascularization and nerve ingrowth, which not only impede the linear alignment and mature remodeling of collagen fibers but also tend to promote fibrosis and chronic pain (Hammerman et al., 2024). The sustained high expression of VEGF-A in the model group at 4 weeks likely reflects a prolonged and unresolved healing pattern, stalled in an immature state. In contrast, the downregulation of VEGF-A in the Tan IIA group at the same time point suggests that its effect is not to inhibit repair, but rather to promote a dynamic optimization of the healing rhythm: promoting angiogenesis in the early phase to support tissue repair, while preventing disorganized vascularization in the later phase by reducing VEGF-A, thus creating a more optimal environment for orderly collagen remodeling and mechanical recovery. This interpretation is not only consistent with findings from previous studies (Korntner et al., 2019; Kirchhoff, 2021) but also aligns with the parallel collagen arrangement shown by Masson staining and the significantly improved biomechanical strength observed in our study, further supporting the positive significance of VEGF-A downregulation during the remodeling phase. This also validates the key regulatory role of the HIF-1 and VEGF signaling pathways in the action of Tan IIA, as predicted by network pharmacology, highlighting the precision and timeliness of its therapeutic intervention.

Inflammation is a core mechanism regulating the early stages of tissue healing. As a major pro-inflammatory factor, IL-6 is upregulated during the acute phase of injury to facilitate inflammatory cell recruitment and necrotic tissue clearance. However, its sustained high expression can delay the repair process and induce a chronic inflammatory response (Soliman and Barreda, 2022). Conversely, IL-10 and TGF-β, as typical anti-inflammatory and reparative factors, help suppress inflammation and induce tissue regeneration (Wong et al., 2025; Sanjabi et al., 2009). Our study found that Tan IIA significantly reduced IL-6 expression while concurrently increasing IL-10 and TGF-β levels. This suggests that Tan IIA may create a favorable condition for tissue repair by reshaping the local inflammatory microenvironment and alleviating excessive inflammation. This phenomenon is closely related to the PI3K-Akt and MAPK pathways predicted by network pharmacology, as these pathways are key nodes in regulating the production and downstream signaling of IL-6 and IL-10 and directly control fibroblast proliferation and function (Deng et al., 2023). It is noteworthy that TGF-β exhibits a dynamic regulatory pattern across different repair stages. It plays a complex dual role, promoting inflammation and proliferation in the early phase while dominating matrix synthesis and maturation in the later phase. At the 4-week time point in our study, the model group had the lowest TGF-β level, possibly indicating that its repair capacity was becoming exhausted in the chronic phase. The ability of Tan IIA to restore its level suggests that Tan IIA may sustain the synthesis of matrix proteins like COL I (consistent with IHC results) by restoring or maintaining a physiological level of TGF-β signaling, thereby pushing the repair process towards a more favorable tissue maturation phase rather than a fibrotic scar phase.

In summary, the *in vivo* experimental results of this study form a strong chain of evidence with the network pharmacology predictions. Tan IIA not only directly regulates key ECM proteins (COL I, VEGFA) and the inflammatory microenvironment (IL-6, IL-10, TGF-β) but also acts in a manner highly consistent with the core pathways predicted by network pharmacology (PI3K-Akt, MAPK, TGF-β). Although this study validated the repair-promoting effects of Tan IIA from multiple perspectives, including tissue structure, collagen expression, and cytokine levels, it has several limitations. First, this study exclusively used male rats in its initial phase to avoid confounding effects from hormonal fluctuations during the female estrous cycle, which can influence key processes such as inflammation, collagen deposition, and angiogenesis, thereby minimizing biological variability. We acknowledge that this introduces a limitation regarding the generalizability of our findings. Future research will address this by conducting comparative experiments between sexes and utilizing ovariectomized models to thoroughly investigate sex differences and the specific role of estrogen, thereby enhancing the clinical translational value of our research. Second, the study was limited to a single endpoint at 4 weeks post-surgery. Although this time point captures the critical transition from the proliferative to the remodeling phase of tendon healing, it is insufficient to fully elucidate the dynamic effects of Tan IIA across all stages—inflammation, proliferation, and long-term remodeling. Future studies should incorporate a systematic, multi-time-point evaluation at early (1–2 weeks), mid (4 weeks), and late (8–12 weeks) phases to more completely characterize its mechanisms of action and enhance the clinical extrapolation value of the results. Third, a significant limitation is the lack of direct validation of the activation status of key signaling pathways, such as PI3K-Akt, MAPK, and TGF-β, at the protein and mRNA levels. Future work will employ techniques such as Western blot, RT-qPCR, and pathway inhibitor experiments to systematically verify the roles of these pathways, thereby further elucidating the molecular mechanisms by which Tan IIA promotes Achilles tendon repair and addressing this gap. Fourth, this study did not perform an *a priori* sample size estimation, which constitutes a methodological limitation in its experimental design. Future research will conduct an *a priori* power analysis based on the means and standard deviations obtained in this study to determine sample sizes more precisely and enhance design rigor. Fifth, although multiple levels of outcome measures were assessed, a global multiple comparison correction was not applied across these different types of indicators. While the high degree of consistency in the results across various dimensions enhances the reliability of the conclusions, we cannot entirely rule out the possibility of an increased risk of Type I error due to multiple testing. Sixth, the biomechanical evaluation focused on ultimate tensile load as the primary indicator of functional recovery. However, we did not report other parameters such as stiffness, strain at failure, or energy to failure, which would have provided a more comprehensive characterization of the repaired tissue’s material properties. For instance, the absence of stiffness data prevents a deeper analysis of the specific effects of different therapies on the maturity and elasticity of the nascent collagen matrix. Future studies should incorporate a more comprehensive assessment of biomechanical parameters to delineate the quality of tissue remodeling with greater detail.

## 5 Conclusion

This study, combining network pharmacology prediction with animal model experiments, confirms that Tan IIA is a key active component of Salvia miltiorrhiza in promoting Achilles tendon repair. Through the construction of a protein-protein interaction network of Tan IIA’s targets and KEGG pathway enrichment analysis, we established that Tan IIA possesses multi-target and multi-pathway intervention characteristics. *In vivo* experiments further demonstrated that Tan IIA can significantly improve the histological structure of the injured tendon, enhance the orderly alignment and mechanical properties of collagen, and modulate the inflammatory microenvironment by promoting the upregulation of anti-inflammatory factors and the downregulation of pro-angiogenic factors, thereby accelerating the overall repair process. The potential mechanism involves the synergistic regulation of key signaling pathways such as TGF-β, MAPK, and PI3K-Akt, which modulate ECM remodeling, cell proliferation, and the inflammatory response. This provides a theoretical basis for further in-depth mechanistic studies. Future research should focus on validating its time-course effects and the key proteins in these signaling pathways to advance its translational application in the repair of tendon injuries.

## Data Availability

The original contributions presented in the study are included in the article/supplementary material, further inquiries can be directed to the corresponding authors.
